# Redox Nanoparticle Therapeutics for Acetaminophen-Induced Hepatotoxicity in Mice

**DOI:** 10.1155/2016/4984597

**Published:** 2016-03-17

**Authors:** Phetcharat Boonruamkaew, Pennapa Chonpathompikunlert, Yukio Nagasaki

**Affiliations:** ^1^Department of Physiology, Faculty of Science, Prince of Songkla University, Hat Yai, Songkhla 90112, Thailand; ^2^Department of Materials Sciences, Graduate School of Pure and Applied Sciences, University of Tsukuba, Tennodai 1-1-1, Tsukuba, Ibaraki 305–8573, Japan; ^3^Master's School of Medical Sciences, Graduate School of Comprehensive Human Sciences, University of Tsukuba, Tennodai 1-1-1, Tsukuba 305–8573, Japan; ^4^Satellite Laboratory, International Center for Materials Nanoarchitectonics (WPI-MANA), National Institute for Materials Science (NIMS), University of Tsukuba, Tennodai 1-1-1, Tsukuba 305–8573, Japan

## Abstract

The purpose of this study was to evaluate the hepatoprotective effect of an antioxidative nanoparticle (RNP^N^) recently developed against APAP-induced hepatotoxicity in mice. The effects of oral administration of RNP^N^ to APAP-treated mice were assessed for various biochemical liver function parameters: alanine transaminase (ALT) activity, aspartate transaminase (AST) activity, alkaline phosphatase (ALP) activity, prothrombin time, and serum albumin (ALB) level. The treatment effects were assessed in terms of free radical parameters: malondialdehyde (MDA) accumulation, glutathione peroxidase (GPx) activity, % inhibition of superoxide anion (O_2_^−∙^), and histopathological examination. The* N*-acetylcysteine (NAC)-treated group exhibited an enhanced prothrombin time relative to the control group, while RNP^N^ did not prolong prothrombin time. The RNP^N^-treated animals exhibited lower levels of ALT, AST, and ALP, while increased ALB levels were measured in these animals compared to those in the other groups. The RNP^N^-treated animals furthermore exhibited improved MDA levels, GPx activity, and % inhibition of O_2_^−∙^, which relate to oxidative damage. Histological staining of liver tissues from RNP^N^-treated animals did not reveal any microscopic changes relative to the other groups. The findings of this study suggest that RNP^N^ possesses effective hepatoprotective properties and does not exhibit the notable adverse effects associated with NAC treatment.

## 1. Introduction

Overdoses of acetaminophen (paracetamol, APAP, or* N*-acetyl-*p*-aminophenol) represent one of the most common pharmaceutical product poisonings in the world [1, 2]. Acetaminophen is widely used as an analgesic and antipyretic drug; however, it is also the leading major cause of acute liver failure (ALF) and acetaminophen overdose may lead to liver transplantation being required or even to death, since the early signs and symptoms of APAP-induced hepatotoxicity are not clear [3–8]. The main mechanism of acetaminophen-induced hepatotoxicity is an increase in oxidative stress and subsequent saturation of the glucuronidation and sulfation pathways of hepatic elimination, leading to more APAP being metabolized to* N*-acetyl-*p*-benzoquinone imine (NAPQI). Finally, GSH depletion (~70–80%) occurs and NAPQI binds to liver cells, resulting in hepatotoxicity [9, 10].

*N*-Acetylcysteine (NAC), a precursor of glutathione (GSH), is the standard antidote administered to patients with APAP-induced hepatotoxicity [11, 12]; however, NAC has previously been shown to have adverse side effects including bruising, bleeding, nausea, vomiting, and diarrhea or constipation. Rarely, NAC also causes rashes, fever, headache, drowsiness, low blood pressure, and liver problems, while NAC therapy was also shown to prolong prothrombin time and should thus be avoided in patients with coagulation disorders [13, 14]. Since NAC is a low molecular weight compound, it furthermore displays a low stability and nonspecific distribution* in vivo* physiological environments, resulting in a low therapeutic efficacy.

Recently, the development of nanotherapeutic strategies against APAP-induced liver injury has been the focus of several research studies [15–18]. We have developed a novel antioxidative nanoparticle, RNP^N^, prepared by self-assembly of an amphiphilic block copolymer, methoxy-poly(ethylene glycol)-b-poly[4-(2,2,6,6-tetramethylpiperidine-1-oxyl) aminomethylstyrene] (PEG-b-PMNT), which has nitroxide radicals on the side chain of the hydrophobic segment. Due to its amphiphilic nature, PEG-b-PMNT forms core-shell-type polymeric micelles with tens of nanometers in size in aqueous media [19]. We previously confirmed that the PEG-b-PMNT polymer internalized and circulated in the blood stream after oral administration of RNP^N^ and is subsequently disintegrated by the gastric acidity [20]. Since the nitroxide radicals in the PMNT segment catalytically eliminate reactive oxygen species (ROS), it functions as a strong antioxidant. However, due to the high molecular weight of the PEG-b-PMNT polymer, it is only minimally internalized in healthy cells, thereby avoiding marked disturbances to normal redox reactions such as those in the electron transport chain, which is adversely affected by conventional low molecular weight antioxidants [21]. On the basis of our previous investigations, therefore, the effects of RNP^N^ on APAP-induced liver injury must be investigated. We report here on both the therapeutic effect and the lack of adverse effects of orally administered RNP^N^.

## 2. Materials and Methods

### 2.1. Materials

1,1,3,3-Tetramethoxypropane (TMP), L-glutathione reduced, glutathione peroxidase (GPx), glutathione reductase (GR), xanthine, xanthine oxidase (XO), *β*-nicotinamide adenine dinucleotide 2′-phosphate reduced tetrasodium (*β*-NADPH) salt, APAP, and NAC were purchased from the Sigma Chemical Company, St. Louis, MO, USA. Other chemical reagents were analytical grade and milli-Q water was used all through the experiments.

### 2.2. Preparation and Characterization of RNP^N^

**Figure 1:**
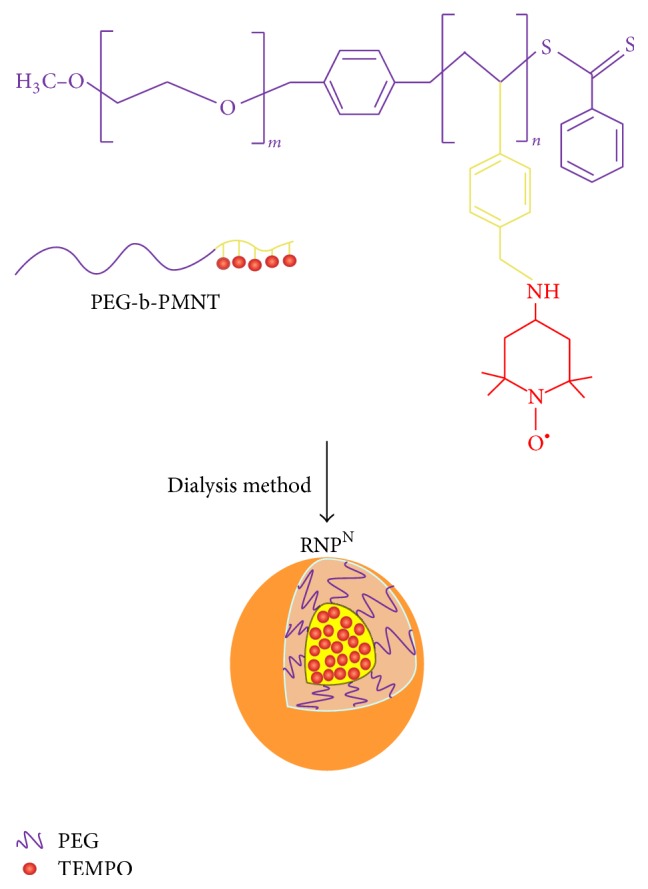
Schematic illustration of antioxidative RNP^N^ preparation.

PEG-b-PMNT was synthesized as previously described [19]. The molecular weight of the PEG segment and the degree of polymerization of PMNT were determined as 5,000 and 13, respectively. The RNP^N^ was prepared by self-assembly of the PEG-b-PMNT using the dialysis method (Figure 1) and the blank micelle (nRNP) was prepared in the same way using PEG-b-poly(chloromethylstyrene), a precursor of PEG-b-PMNT with the same molecular weight. The size and distribution of the resulting RNP^N^ and the nRNP in aqueous solution were determined by dynamic light scattering (DLS) measurements carried out in triplicate. Electron paramagnetic resonance (EPR) was used to quantify the amount of 2,2,6,6-tetramethylpiperidine-1-oxyl (TEMPO) inside each RNP^N^.

### 2.3. Animal Care Conditions

Animal experiments were carried out using male CD-1® IGS mice nomenclature: Crl:CD1(ICR) (10 weeks old, 40–50 g) purchased from Charles River Japan, Inc. (Yokohama, Japan). The mice (*n* = 48) were maintained in the Laboratory Animal Resource Center, University of Tsukuba (Japan), in six groups. The animals were housed in stainless steel cages and seven days prior to the experiment they were acclimated to the standard laboratory conditions of 23 ± 1°C, ventilation with relative humidity of 50 ± 5%, and a 12 h light/12 h dark cycle. The mice received food and filtered water* ad libitum *throughout the study. All animal experiments were undertaken in accordance with the criteria outlined in the license (numbers 15–434) approved by the Animal Ethics Committees of the University of Tsukuba.

### 2.4. APAP-Induced Hepatotoxicity Model

Mice were randomly divided into six groups of 8 animals each:

 Group A: control, no treatment.
 Group B: 0.9% normal saline (NSS) + APAP 2.5 g/kg body weight (BW)
   (the APAP concentration has been used according to our pilot study).
 Group C: N-acetylcysteine (NAC) 600 mg/kg BW + APAP 2.5 g/kg BW.
 Group D: low molecular weight (LMW) TEMPO 15.4 mM + APAP 2.5 g/kg BW.
 Group E: blank micelle (nRNP) 12.5 mg/mL + APAP 2.5 g/kg BW.
 Group F: RNP^N^ 12.5 mg/mL (containing 4-amino TEMPO 15.4 mM) + APAP 2.5 g/kg BW.

**Figure 2:**
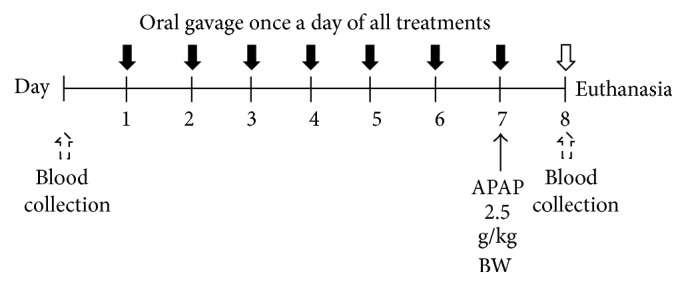
Experimental design of APAP-induced hepatotoxicity model.

Prior to the APAP-induced hepatotoxicity experiments, blood was collected from the tail veins of all mice to analyze prothrombin time. All mice in the treatment groups were orally administered their treatments once daily for 7 days after which hepatotoxicity was induced by oral gavage of 2.5 g/kg BW of APAP (Figure 2). After 24 h, the mice were rechecked of prothrombin time and anesthetized with intraperitoneal (i.p.) injection of pentobarbital 50 mg/kg BW. The blood was drawn and treated with anticoagulant to allow serum by centrifugation of the blood at 1600 g for 15 min at 4°C in order to determine the liver function. Animals were then sacrificed and their livers were removed. Half of each liver was fixed in 10% buffered formalin for histopathology analysis and the other half was homogenized (10% w/v) in ice cold phosphate buffer (50 mM, pH 7.4). The liver homogenates were centrifuged at 9,000 g for 15 min at 4°C and the resulting supernatants were frozen at −80°C until being subjected to oxidative stress analysis and lipid peroxidation assay.

### 2.5. Lipid Peroxidation (LPO) Assay

The levels of malondialdehyde (MDA), the product of lipid peroxidation, were assessed based on the method described by Ohkawa et al. [22]. Briefly, the samples were treated with thiobarbituric acid (TBA) to allow for thiobarbituric acid reactive substance (TBARS), a pink complex and indicator of lipid peroxidation, to be quantified. Absorbance levels at 532 nm were measured by spectrophotometer and the arbitrary values obtained were related to MDA concentration (nmol/g tissue) using a standard curve generated from the absorbance values obtained for standard solutions of 1,1,3,3-tetramethoxypropane (TMP).

### 2.6. Glutathione Peroxidase (GPx) Assay

GPx activity was determined according to the method described by Hussain et al. [23]. Glutathione peroxidase (GPx) is a radical-scavenging enzyme that catalyzes the reduction of hydrogen peroxide (H_2_O_2_) and lipid peroxide (ROOH) using glutathione (GSH), resulting in the formation of oxidized glutathione (GSSH) and H_2_O. Glutathione reductase (GR) subsequently catalyzes GSSH by reacting with nicotinamide adenine dinucleotide phosphate (NADPH) yielding GSH and NADP^+^. The generation of NADP^+^ was measured spectrophotometrically at 340 nm relative to a blank sample and a standard curve was generated from GPx solutions. GPx activity was expressed as units/mg protein, where protein concentration was measured using the method of Lowry et al. [24].

### 2.7. Superoxide (O_2_^−∙^) Anion Assay

The O_2_^−∙^ levels in the samples were determined by spectrophotometric measurement based on the xanthine/xanthine oxidase (XO) system involving the conversion of yellow nitro blue tetrazolium (NBT) to blue formazan. Reactions contained EDTA, NBT, xanthine, XO, and samples were assessed at 560 nm relative to a standard curve generated using 4-hydroxy-2,2,6,6-tetramethylpiperidin-1-oxyl (TEMPOL). Data were expressed as % inhibition of O_2_^−∙^ [25].

### 2.8. Biochemical Assays in Plasma

Blood for biochemical analysis was collected of 100 *μ*L from the animals prior to treatment via the lateral tail vein and 500 *μ*L after APAP-induced hepatotoxicity induction through heart. The blood was centrifuged (1600 g, 15 min, 4°C) to isolate the serum, which was subsequently analyzed with a FUJI DRI-CHEM 7000V (Fujifilm, Japan) for biochemical liver activities of albumin (ALB), alkaline phosphatase (ALP), aspartate aminotransferase (AST), and alanine aminotransferase (ALT).

### 2.9. Liver Histopathology

The liver tissues fixed in 10% neutral buffered formalin solution were cleaned in running water before being processed for further histopathological examination. Paraffin-embedded tissues were sectioned (5 *μ*m thickness), stained with hematoxylin and eosin (H&E), assessed for histopathological changes by light microscopy, and graded blindly and independently using the modified method described by Wood et al. [26]. Grades (0–5) were as follows: 0, no lesions; 1, minimal lesions, only necrotic cells at the first cell level from the central vein; 2, mild lesions, necrotic cells extending two to three cell levels from the central vein; 3, moderate lesions, necrotic cells extending three to six cell levels within peripheral distribution; 4, marked lesions, the same as in 3 but with necrosis extruding to another central vein; and 5, lesions more severe than those in 4, with progressive centrilobular necrosis throughout the section.

### 2.10. Statistical Analysis

Data are expressed as mean ± SEM. Groups were compared by one-way analysis of variance (ANOVA) followed by a post hoc multiple comparison test (Tukey's method) using SPSS version 16.0. Differences with *p* < 0.05 were considered statistically significant.

## 3. Results

### 3.1. The Size and Distribution of RNP^N^ and nRNP

**Figure 3:**
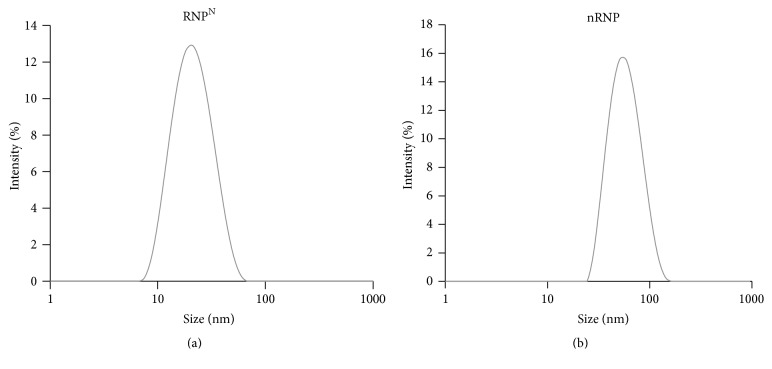
Size distribution of RNP^N^ (a) and nRNP (b) assessed by dynamic light scattering (DLS).

The DLS measurements confirmed the unimodal distribution of nanoparticles in the nanometer size range with no particle aggregation (Figure 3). The particle size and distribution were found to be 22.140 ± 0.307 nm and 0.139 ± 0.008 for RNP^N^ and 58.853 ± 0.696 nm and 0.205 ± 0.004 for nRNP, respectively. From the EPR spectrum of RNP^N^, the amount of nitroxide radical in RNP^N^ (12.5 mg/mL) was 15.4 mM.

### 3.2. Effects of RNP^N^ on Serum ALB, ALP, ALT, and AST Levels

**Figure 4:**
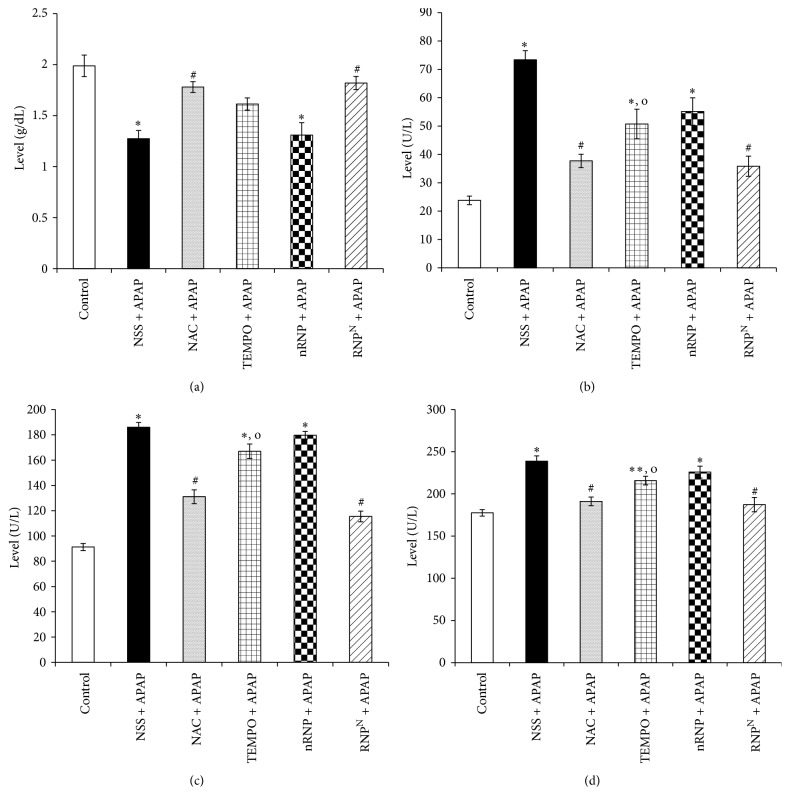
Biochemical liver function in terms of (a) ALB, (b) ALT, (c) AST, and (d) ALP activities after treatment for 7 days and APAP-induced hepatotoxicity. Data were expressed as mean ± SEM, ^*∗*^versus control group, *p* < 0.001; ^*∗∗*^versus control group, *p* < 0.01; ^#^versus NSS + APAP-treated group, *p* < 0.001; ^o^versus  RNP^N^ + APAP-treated group, *p* < 0.05; *n* = 8 mice/group.

Hepatotoxicity was determined by quantitative analysis of ALB, ALT, AST, and ALP levels as shown in Figure 4. Serum ALB, ALT, AST, and ALP levels in control mice were 1.9875 ± 0.2997 g/dL, 23.75 ± 4.301 U/L, 91.25 ± 7.995 U/L, and 177.5 ± 10.915 U/L, respectively. The NSS + APAP-treated group exhibited decreased ALB levels, while the ALT, AST, and ALP levels in the serum of the animals in this group were significantly elevated compared to those measured for the control group (*p* < 0.001). Interestingly, the ALT, AST, and ALP levels in the RNP^N^ + APAP-treated animals did not differ from those in the NAC + APAP-treated group but differed significantly from those in the NSS + APAP-treated group (*p* < 0.001), indicating that RNP^N^ suppressed the adverse effects of APAP in mice. Despite the same dose of nitroxide radicals being administered in both groups, the protective effect of RNP^N^ was stronger than that of the low molecular weight (LMW) TEMPO, especially in terms of ALT, AST, and ALP levels.

### 3.3. Effects of Treatments on Prothrombin Time

**Figure 5:**
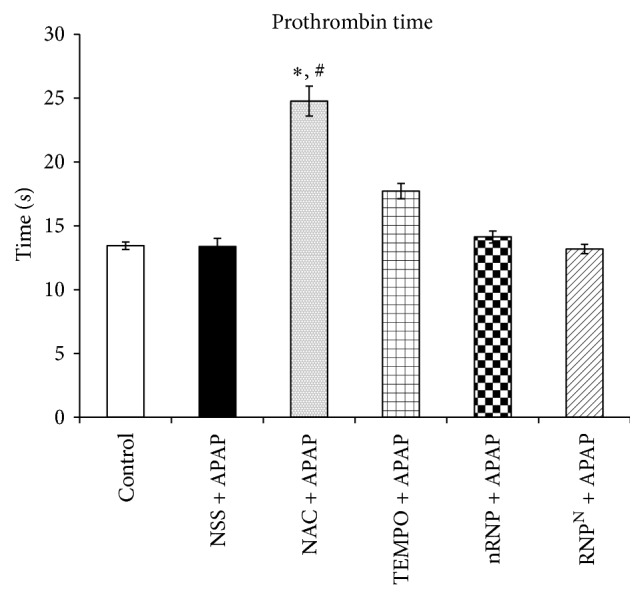
Effects of treatments on prothrombin time after treatment for 7 days. Data represent mean ± SEM, ^*∗*^versus control group, *p* < 0.001; ^#^versus NSS + APAP-treated group, *p* < 0.001; *n* = 8 mice/group.

All blood samples were tested for prothrombin time (Figure 5). The NAC + APAP-treated animals exhibited a significantly prolonged prothrombin time (24.778 ± 3.308 s) compared with the prothrombin times measured in the control (13.444 ± 0.882 s) and NSS + APAP (13.375 ± 1.84 s) group animals (*p* < 0.001). Prothrombin time, one of the major side effects of NAC, results in hepatocellular necrosis or disseminated intravascular coagulation (DIC). Interestingly, RNP^N^ + APAP treatment did not prolong prothrombin time despite exhibiting strong therapeutic effects as stated above.

### 3.4. Effects on MDA Levels, GPx Activity, and % Inhibition of O_2_^−∙^

**Figure 6:**
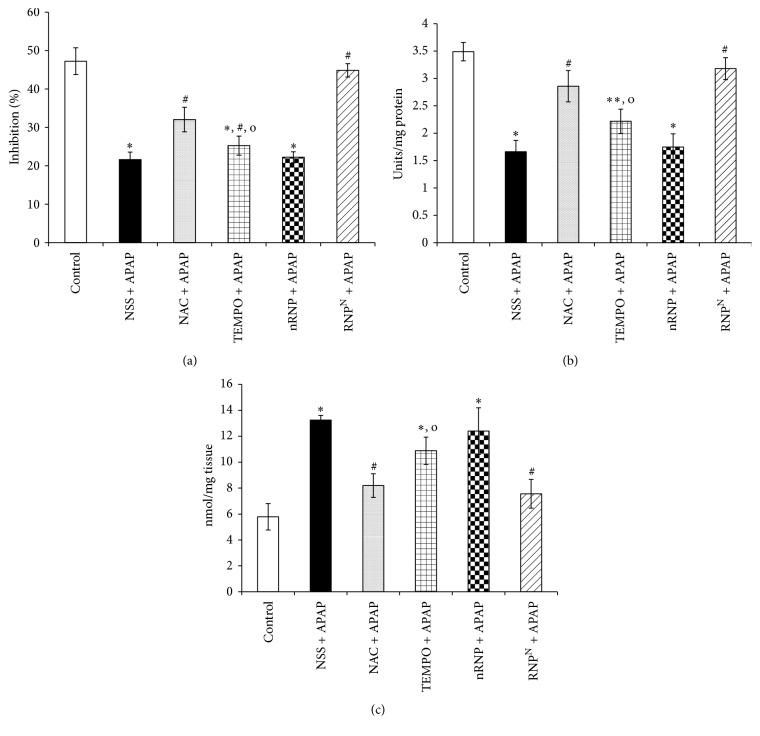
Effects of treatment for 7 days on (a) % inhibition of O_2_^−∙^, (b) GPx activity, and (c) MDA levels. Data represent mean ± SEM, ^*∗*^versus control group, *p* < 0.001; ^*∗∗*^versus control group, *p* < 0.01; ^#^versus NSS + APAP-treated group, *p* < 0.01; ^o^versus  RNP^N^ + APAP-treated group, *p* < 0.05; *n* = 8 mice/group.

The levels of MDA, the GPx activity, and the % inhibition of O_2_^−∙^ measured in the livers of all groups are presented in Figure 6. Liver MDA levels were found to be elevated in the NSS + APAP group compared with the control group, while GPx activity and % inhibition of O_2_^−∙^ were lower in the NSS + APAP group compared with the control group (13.251 ± 0.365 nmol/mg tissue versus 5.784 ± 1.015 nmol/mg tissue, 1.660 ± 0.207 units/mg protein versus 3.489 ± 0.168 units/mg protein, and 21.604 ± 1.969% versus 47.237 ± 3.505%, resp.; *p* < 0.001). Remarkably, RNP^N^ + APAP induced a noticeable decrease in MDA levels relative to control levels, whereas GPx activity and % inhibition of O_2_^−∙^ were found to be elevated in the RNP^N^ + APAP group compared with the control group (7.558 ± 1.101 nmol/mg tissue versus 13.251 ± 0.365 nmol/mg tissue, 3.178 ± 0.201 units/mg protein versus 1.660 ± 0.207 units/mg protein, and 44.881 ± 1.749% versus 21.604 ± 1.969%, resp.; *p* < 0.01). No significant differences in the MDA levels, the GPx activity, and the % inhibition of O_2_^−∙^ were observed between the RNP^N^ + APAP- and NAC + APAP-treated groups. These findings again demonstrate the protective effect of RNP^N^ to be significantly stronger than that of LMW TEMPO.

### 3.5. Histopathological Examinations of APAP-Induced Liver Toxicity

**Figure 7:**
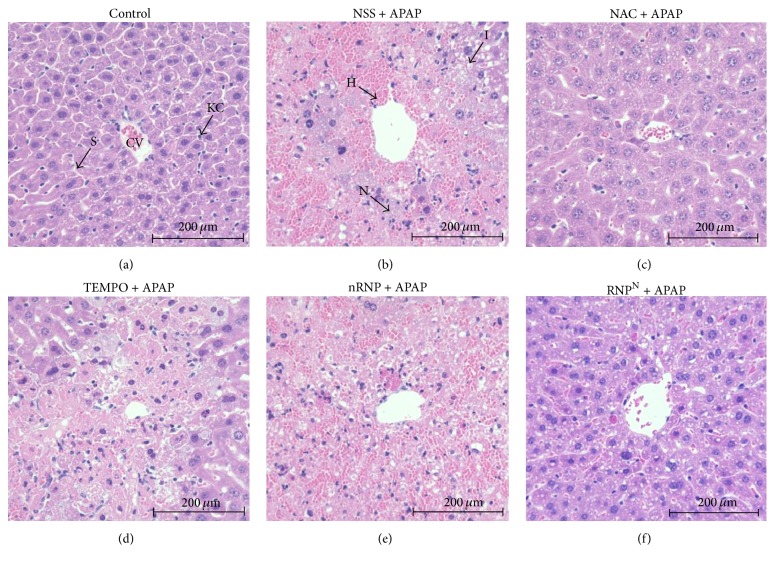
Hepatoprotective effect of treatments in mice with APAP-induced liver damage. (a) control group. (b) APAP-induced group. ((c)–(f)) APAP group treated with (c) 600 mg/kg BW of NAC, (d) 15.4 mM of LMW TEMPO, (e) 12.5 mg/mL of nRNP, (f) and 12.5 mg/mL of RNP^N^. Liver sections were stained with hematoxylin and eosin (×400). CV: central vein; S: sinusoids; KC; Kupffer cell; N: necrosis; I: inflammation; H: hemorrhage.

**Figure 8:**
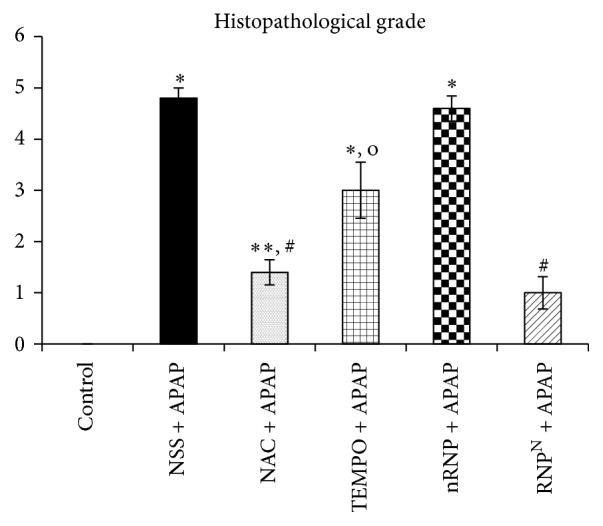
Histopathological changes in liver tissue due to APAP-induced hepatotoxicity. Data represent mean ± SEM, ^*∗*^versus control group, *p* < 0.001; ^*∗∗*^versus control group, *p* < 0.05; ^#^versus NSS + APAP-treated group, *p* < 0.001; ^o^versus  RNP^N^ + APAP-treated group, *p* < 0.001; *n* = 5 samples/group.

After seven days of treatment, liver tissues were taken from the animals of all groups and were subjected to histological analysis (Figure 7). The livers from the animals in the control, NAC + APAP, and RNP^N^ + APAP groups had an overall smooth appearance and normal color. The control group livers were further found to have normal lobular morphology and hepatocytes with well-defined sinusoids (Figure 7(a)). The hepatic injury in the mice treated with APAP (2.5 mg/kg BW dissolved in normal saline) manifested as inflammatory infiltration, swelling, hemorrhage, and necrosis involving mainly the centrilobular zone (Figure 7(b)). Mild congestion of sinusoidal spaces was observed in the centrilobular area of the livers of NAC + APAP-treated mice (Figure 7(c)). The livers of the mice treated with LMW TEMPO and nRNP appeared hyperemic, mottled and were fragile (Figures 7(d) and 7(e)). Remarkably, the livers of the mice treated with RNP^N^ did not reveal any significant microscopic changes relative to control tissue (Figure 7(f)). These findings are in agreement with the histopathological scale analysis of the liver sections (Figure 8): the RNP^N^-treated group exhibited significantly less severe hepatic injury than the NSS, TEMPO, and blank micelle-treated groups (*p* < 0.001).

## 4. Discussion

The nanoparticle is one of the novel drug carriers for therapeutic and diagnostic objectives which has several potential effects in improving accumulation and bioavailability of drug in target side thereby suppressed immunogenicity or drug resistance and finally reducing adverse effects. Additionally, nanoparticles also promote drug solubility, controlled and sustained drug release, decreased drug elimination, and delivered more drugs combination treatment for synergistic effect [27–29].

Liver injury induced by APAP is one of the most causes of ALF worldwide and the mortality rate of ALF is ~20–40% [3, 4]. Characteristics of APAP-induced ALF include high levels of liver ALT, AST, and ALP enzymes after 24–72 h [30, 31]. Here, we show that NSS + APAP treatment raises the levels of ALT, AST, and ALP, while lowering the level of ALB, indicating successful preparation of the APAP-induced hepatotoxicity model. Treatment with NAC or RNP^N^, on the other hand, was shown to suppress these APAP-induced increases. Treatment with LMW TEMPO and nRNP did not have the same suppressive effect. In addition, our previous research finding found that RNP^N^ at dose of 300 mg/kg BW via oral administration for 1 month in mice did not show any toxicity in several organs including liver [20].

At therapeutic doses, about 90% of APAP is eliminated via sulfation or glucuronidation pathways [32, 33] and another 5% is metabolized by cytochrome P450 2E1 (CYP2E1) to NAPQI [34, 35]. The NAPQI subsequently binds to glutathione (GSH) to produce mercuric acid and cysteine conjugates before being eliminated from the body [11]. An overdose of APAP may result in the depletion of GSH and cause NAPQI-induced hepatic cell injury [11, 36–39]. Our GPx activity results showed that RNP^N^ treatment increased GPx activity compared to treatment with NSS, TEMPO, or nRNP, indicating the elevation of GSH level like NAC-treated group.

The production of ROS including hydrogen peroxide, hydroxyl radicals, and superoxide anions can be enhanced by NAPQI. Lipid peroxidation, DNA, and protein oxidation, as well as a decrease in radical-scavenging enzymes of GPx and superoxide dismutase (SOD) have also been reported in APAP-induced liver injury [40–42]. This mechanism has been proposed as a key player in the oxidative stress and hepatic injury in APAP-induced hepatotoxicity [39–43] and we therefore measured lipid peroxidation, and % inhibition of O_2_^−∙^ in this study to confirm ROS production in our APAP-induced hepatotoxicity model. Interestingly, treatment with RNP^N^ was found to diminish lipid peroxidation compared to treatment with NSS, TEMPO, or nRNP, while % inhibition of O_2_^−∙^ was also higher in the RNP^N^-treated group compared with the NSS-, NAC-, TEMPO-, and nRNP-treated groups. Although LMW TEMPO, like RNP^N^, possesses ROS scavenging activity, the scavenging efficiency of the LMW TEMPO is lower than that of the RNP^N^, probably due to the rapid elimination, easy metabolism, and marked disturbance of normal redox reaction in normal cells. We have previously reported that only 5–7% of PEG-b-PMNT internalizes in blood stream by oral administration [20]. Prolonged circulation tendency of the internalized PEG-b-PMNT in blood stream might improve an access to liver tissue, which might improve therapeutic efficiency as compared with LMW TEMPO.

The standard treatment for APAP-induced liver toxicity is NAC, which acts as a GSH precursor to increase the GSH reservoir. Treatment with NAC, however, triggers an impaired coagulation cascade, which is the reason why prothrombin time was assessed in this study. Mice in the NAC + APAP-treated group were shown to have significantly prolonged prothrombin times compared to other groups (*p* < 0.001), while the mice in the RNP^N^ + APAP-treated group did not exhibit this side effect.

The findings from the hepatic function analyses in this study were confirmed by the histopathological changes observed by microscopic analysis. In the NSS + APAP, TEMPO + APAP, and nRNP + APAP groups, inflammatory infiltration of lymphocytes, vacuolation, swelling, and centrilobular necrosis were observed, while pretreatment with RNP^N^ and NAC was found to prevent these histological changes. Mice in the NAC + APAP-treated group, however, exhibited mild changes in hepatocyte and sinusoid arrangement. Furthermore, the previous study showed that the RNP^N^ treatment ameliorates nonalcoholic steatohepatitis NASH fibrosis via the decrease of hepatic stellate cell activation marker of alpha-smooth muscle actin (*α*-SMA) [44].

We report here that RNP^N^ successfully ameliorated APAP-induced hepatotoxicity in mice as demonstrated by decreased levels of the hepatic injury markers ALT, AST, and ALP and increased ALB levels. The observed protective effects of RNP^N^ may be due to antioxidant effects as shown by the reduced lipid peroxidation, increased GPx activity, and increased % inhibition of O_2_^−∙^. The histopathological analysis conducted in this study did not reveal any toxicity by RNP^N^. Therefore, the patients who are long-term users of APAP need to suppress liver damage. So, combination of APAP with RNP^N^ is also very interesting strategy to suppress hepatotoxicity.

## 5. Conclusion

The findings of this study indicate that RNP^N^ has a hepatoprotective effect against APAP-induced liver injury via antioxidant properties decreasing lipid peroxidation while increasing GPx activity and the % inhibition of O_2_^−∙^. Treatment with RNP^N^ furthermore shows no side effect of coagulation cascade impairment, indicating that RNP^N^ may be a more effective treatment for APAP-induced hepatotoxicity than NAC. Our findings lead us to conclude that antioxidative nanomedicine is a promising strategy for improving therapeutic effects by suppressing disturbances of normal redox reactions in healthy cells.
